# Differential Corticomotor Excitability Responses to Hypertonic Saline-Induced Muscle Pain in Forearm and Hand Muscles

**DOI:** 10.1155/2018/7589601

**Published:** 2018-04-22

**Authors:** Dennis B. Larsen, Thomas Graven-Nielsen, Rogerio P. Hirata, Shellie A. Boudreau

**Affiliations:** ^1^Center for Neuroplasticity and Pain (CNAP), SMI, Aalborg University, Aalborg, Denmark; ^2^SMI, Aalborg University, Aalborg, Denmark

## Abstract

Experimental muscle pain inhibits corticomotor excitability (CE) of upper limb muscles. It is unknown if this inhibition affects overlapping muscle representations within the primary motor cortex to the same degree. This study explored CE changes of the first dorsal interosseus (FDI) and extensor carpi radialis (ECR) muscles in response to muscle pain. Participants (*n* = 13) attended two sessions (≥48 hours in-between). Hypertonic saline was injected in the ECR (session one) or the FDI (session two) muscle. CE, assessed by transcranial magnetic stimulation (TMS) motor-evoked potentials (MEPs), was recorded at baseline, during pain, and twenty minutes postinjection together with pain intensity ratings. Pain intensity ratings did not differ between the two pain sites (*p* = 0.19). In response to FDI muscle pain, the MEPs of the FDI muscle were reduced at 2 and 4 min postinjection (*p* ≤ 0.03), but not after ECR muscle pain. No significant MEP change was detected for the ECR muscle (*p* = 0.62). No associations between MEPs and pain intensity were found (*p* > 0.2). The present results indicate that the output from overlapping cortical representations of two muscles differentially adapts to acute muscle pain.

## 1. Introduction

Musculoskeletal pain disorders are leading cause of disability in the world [1]. This underscores the importance of targeting underlying mechanisms in the transition from acute-to-chronic musculoskeletal pain. Nonetheless, the mechanisms remain poorly understood and therefore pose a challenge to effectively treat [2]. It has been proposed that central nervous system neuroplasticity may play a pivotal role in the persistence of pain [3] as seen in, for example, low-back [4] and phantom-limb pain patients [5]. In this respect, it is well established that acute muscle pain alters the function of human sensorimotor cortices, often reflected in a moderate-to-strong reduction in sensory and primary motor cortex (MI) excitability, as assessed by somatosensory-evoked potentials or motor-evoked potentials (MEPs), respectively [6]. A current opinion is that following an injury to a segment of the body, the net corticomotor excitability representing the injured segment reduces [7]. The reduction in corticomotor excitability may be a protective mechanism that serves to prevent further damage by inhibiting motor output [7]. Experimental muscle pain studies have indeed demonstrated impaired motor control in response to experimental pain [8, 9]. Additionally, acute muscle pain can induce a strong inhibition of corticomotor excitability of the muscle in pain that persists, despite no ongoing pain, for up to 30 min [10–13]. Such neuroplastic pain-related modulations suggest that corticomotor inhibition serves to protect a painful limb even after pain has dissipated, but also that several muscles may be affected by this inhibition [10, 14].

The body divisions are generally represented in specific areas of the MI giving rise to a topography [15]. However, it is evident that MI topography is not bound to a strict representation of each individual muscle, and this has been shown in detail for the hand [16]. The upper limb muscle representations within the MI are relatively well defined but also show strong overlap between muscles such as the first dorsal interosseous (FDI) and extensor carpi radialis (ECR) muscles [17], which can be further expanded with training [18] or smudged during chronic pain conditions [19]. For instance, a study exploring the representation of upper limb musculature in elite volleyball players demonstrated a greater overlap of the medial deltoid and the ECR muscles, which is activated when performing a volleyball spike, when compared to elite endurance athletes (runners) [18].

What remains unknown is whether the presence of acute muscle pain in one muscle simultaneously inhibits corticomotor excitability of overlapping muscle representations within the MI. Therefore, the current study was aimed at exploring the impact of experimentally induced muscle pain on corticomotor excitability in muscles with known overlapping MI representations, specifically the ECR and the FDI muscles. It was hypothesized that the corticomotor excitability and cortical representations of FDI and ECR muscles would (1) reduce in response to experimental pain induced in the ECR or FDI muscle, and (2) that MI inhibition would outlast the perception of pain, as earlier shown, up to 10 min post pain.

## 2. Methods and Materials

### 2.1. Participants

The effect size (standardized mean difference) of acute experimental pain on corticomotor excitability reduction at rest was based on a recent meta-analysis and systematic review (0.52 (−0.01, 1.06)) [6] and used to estimate the sample size of the current study.

Eighteen healthy, right-handed participants (mean age ± SD: 27.8 ± 6.0; 18–45 years; 7 women) with no recent history of upper limb (acute/chronic pain), musculoskeletal, or neurological conditions were included. Prior to inclusion, all participants were screened using the transcranial magnetic stimulation (TMS) screening questionnaire [20, 21]. All participants were right-handed as assessed by the Edinburgh Handedness Inventory (mean laterality quotient ± SD: 0.90 ± 0.13) [22].

The study was approved by the local ethics committee (VN-20170006) and conducted in accordance with the Declaration of Helsinki. Written informed consent was obtained prior to participation.

### 2.2. Experimental Design

**Figure 1:**
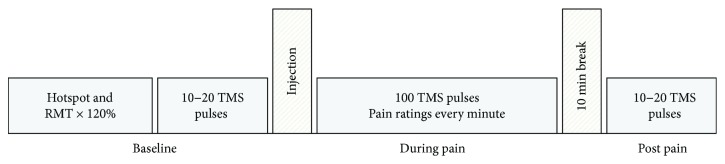
Experimental design. Participants joined two experimental sessions separated by at least 48 hours. In session one, experimental pain was induced by injection of hypertonic saline into the ECR muscle and in session two the FDI muscle was injected. The procedure for both sessions was identical with the only difference being the optimal scalp position determined for either the ECR or FDI muscle. ECR: extensor carpi radialis; FDI: first dorsal interosseus; RMT: resting motor threshold; TMS: transcranial magnetic stimulation.

The experiment was performed over two sessions separated by at least 48 hours where the corticomotor excitability of the ECR and FDI motor representations were assessed in response to experimental ECR muscle pain or FDI muscle pain, one pain condition per session (Figure 1). Participants were seated comfortably in a chair and their arm rested in an approximate 45° angle flexion. Corticomotor excitability was assessed by MEPs evoked by TMS and recorded at baseline. Experimental muscle pain was then induced by injection of hypertonic saline into the ECR or the FDI muscle. Immediately after the injection, participants were asked to rate the pain intensity, followed by pain intensity rating every minute for the next 10 min (during pain). Concurrently, 10 TMS pulses were delivered every minute for 10 min. Following the 10 min of TMS and pain intensity rating, a 10 min break ensued. During this, the participants were instructed to remain relaxed. Twenty minutes postinjection, corticomotor excitability was assessed again.

### 2.3. Transcranial Magnetic Stimulation

The TMS methods are described in accordance with the recent guidelines on TMS methodology reporting [23]. Monophasic TMS pulses were delivered with a magnetic stimulator (Magstim BiStim^2^, Magstim Company, UK), using a focal figure-of-eight coil (D70^2^, Magstim Company, UK). Stimulations were performed with the coil handle pointing backwards and laterally at a 45° angle to the sagittal plane, inducing a posterior-anterior directed current, to elicit MEPs from the ECR and FDI muscles. All TMS pulses were delivered with an interstimulus interval (ISI) of 5–7 seconds.

To standardize orientation and location of TMS pulse delivery, the participants were fitted with a swimming cap containing a predefined grid (1 × 1 cm squares) orientated to the vertex (0, 0). This was used to determine the optimal scalp position (hotspot) and resting motor threshold (RMT) for either the ECR or FDI muscle. The hotspot for the ECR or FDI muscle, dependent on session (i.e., ECR hotspot for the ECR muscle pain session and vice versa for FDI muscle pain), was determined using 50% of maximum stimulator output and was defined as the site that yielded the most consistent and highest peak-to-peak amplitude TMS-MEPs in three trials. Subsequently, the RMT at the hotspot was determined based on the stimulus intensity needed to evoke MEPs ≥ 50 *μ*V in the ECR or FDI muscle in five out of 10 trials with the muscles at rest [24]. The stimulation intensity was set to 120% × RMT for the remaining of the experiment. To ensure that the baseline TMS-MEPs were sufficient to capture the corticomotor excitability, 2 × 10 TMS-MEPs with 30 s break in-between were recorded during the ECR muscle pain session in four participants and all participants for the FDI muscle pain session. The remaining baseline averages are based on 10 TMS-MEPs.

### 2.4. Recording of Motor-Evoked Potentials

Surface electromyography (EMG) was recorded from the muscle belly of the ECR and FDI muscles with bipolar Ag/AgCl electrodes (Neuroline 720, Ambu® A/S, DK) placed with an approximate 20 mm interelectrode distance. The reference electrodes were placed at the styloideus processus (FDI) or the olecranon (ECR), both of the ulna. The EMG data were sampled at 4 kHz, preamplified (1000x gain), and analogue bandpass filtered at 5 Hz–1 kHz. EMG data were digitized by a 16-bit data acquisition card (National Instruments, NI6122; voltage: 10 V), and peak-to-peak TMS-MEPs were shown online by custom-made LabVIEW software (Mr. Kick III, SMI, Aalborg University).

### 2.5. Experimental Muscle Pain

The sites of injections were determined by palpation of the contracted ECR or FDI muscle, and the skin was cleaned with alcohol. A bolus injection of hypertonic saline (5.8% NaCl) was administered to either the ECR or the FDI muscle using a 1 mL syringe with a disposable needle (27G). The volume of the bolus was 0.5 mL for the ECR muscle [25] and 0.2 mL for the FDI muscle [10]. To assess the intensity of pain in response to the hypertonic saline injection, the participants were asked to rate the pain intensity on a 0–10 numerical rating scale (NRS) (“*0*” representing “*no pain*” and “*10*” representing “*worst imaginable pain*”) immediately after injection, every minute during pain, and at 20 min postinjection.

### 2.6. Statistical Analysis

Data are presented as mean MEPs ± standard error of the mean (SEM) unless otherwise stated. Peak-to-peak values of raw MEPs for the ECR and FDI muscles were extracted for both sessions and used for analysis. Each MEP was assessed offline for any motor activity immediately prior to the stimulation, and if present (≥40 *μ*V), excluded from the analysis (a total of 1.5% of all MEPs was rejected based on this premise). MEPs recorded during pain were averaged based on 20 stimulations (i.e., every two min during pain) to reflect corticomotor excitability change over time, yielding a total of seven time points (baseline, five time points during pain, and 20 min postinjection). For each time point, MEPs for the FDI and ECR muscles were subjected to an analysis for normality (Shapiro-Wilk test for normality) and log transformed (residuals were retested for normal distribution). MEPs from each muscle were analysed using a two-way repeated measures' analysis of variance (RM ANOVA) (within-subjects' factors: pain site [ECR pain or FDI pain] × time [baseline, five time points during pain, and 20 min postinjection]). Bonferroni-corrected post hoc analyses were performed as appropriate. NRS scores of the pain intensity were averaged in epochs of 2 min and subjected to a two-way RM ANOVA (within-subjects' factors: pain site [ECR pain or FDI pain] × time [baseline, five time points during pain, and 20 min postinjection]). To clarify if changes in corticomotor excitability and peak pain intensity were associated, the relative change in MEPs at peak pain compared to baseline (MEP_peak pain_/MEP_baseline_) was calculated for each participant and correlated with the corresponding peak pain intensity. Correlation analysis was performed using Spearman's ranked correlation analysis. Statistical analyses were carried out in Statistical Package for Social Sciences (SPSS; version 24, IBM). A *p* value < 0.05 was considered statistically significant.

## 3. Results

### 3.1. Participants and TMS Parameters

Out of the 18 included participants, 14 returned for the FDI pain session (age: 29.6 ± 5.4, 18–45 years). Data from one participant was removed from analysis due to technical issues evoking TMS-MEPs during the FDI pain session. Therefore, the analysis was performed on 13 participants (5 women).

The RMT of the ECR muscle was 43.5 ± 9.6% of the maximum stimulator output and 40.3 ± 8.1% for the FDI muscle. In the FDI pain session, which included two blocks of 10 TMS-MEPs, no significant difference was found between the first and second blocks of ten recorded ECR and FDI MEPs, *p* = 0.34 and *p* = 0.49, respectively, paired sample *t*-test. These results indicate that the recording of 10 MEPs was sufficient to capture the corticomotor excitability at baseline. The amplitude of the baseline MEPs did not significantly differ between sessions for either the ECR or the FDI (both *p* > 0.17, paired sample *t*-tests). Furthermore, the average distance from vertex for the ECR hotspot was 1.5 cm (anterior-posterior) and 5.1 cm (mediolateral), whereas it was 1.7 cm (anterior-posterior) and 5.1 cm (mediolateral) for the FDI hotspot.

### 3.2. Pain Intensity for the ECR Injection versus the FDI Injection

**Figure 2:**
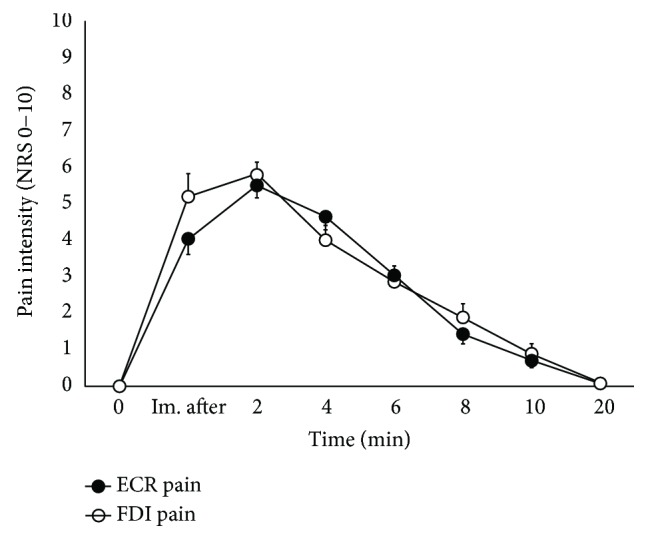
Mean (±SEM) NRS scores of the pain intensity ratings over time for hypertonic saline injection into the ECR (filled circles) or FDI (open circles) muscle. No significant difference was found in the two pain profiles over time. ECR: extensor carpi radialis; FDI: first dorsal interosseus; NRS: numerical rating scale; Im. after: immediately after injection of hypertonic saline.

The NRS scores of the pain intensity following the ECR and FDI muscle injections of hypertonic saline were not significantly different (*F*(1.91, 22.88) = 1.49, *p* = 0.19, *η*^2^_partial_ = 0.11) although there was an overall time effect (*F*(2.44, 29.32) = 76.45, *p* < 0.005, *η*^2^_partial_ = 0.86) demonstrating peak pain intensity at 2 min compared to immediately postinjection (*p* = 0.001) (Figure 2).

### 3.3. Corticomotor Excitability for the ECR and FDI Muscle

**Figure 3:**
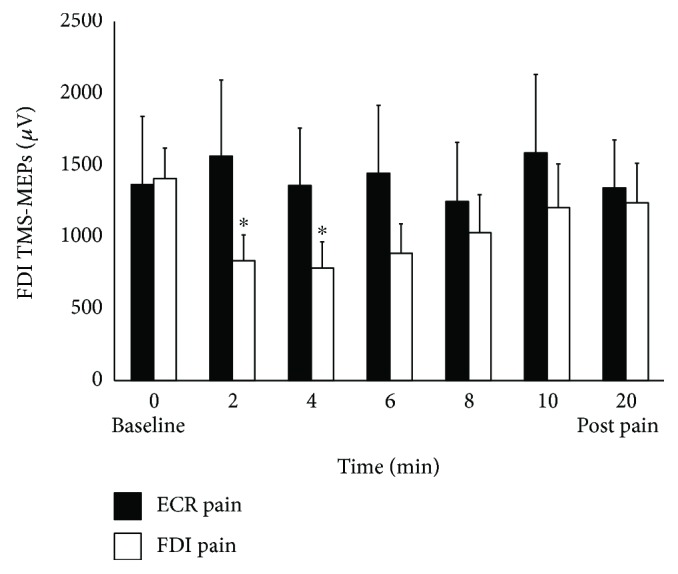
Mean (±SEM) MEPs of the FDI muscle following hypertonic saline injection into the ECR muscle (solid bars) and FDI muscle (open bars). Significantly reduced MEP as compared to baseline is illustrated (^∗^*p* < 0.05; Bonferroni corrected). FDI: first dorsal interosseus; TMS: transcranial magnetic stimulation; MEPs: motor-evoked potentials.

The two-way RM ANOVA for FDI MEPs showed an interaction between pain site and time (*F*(3.002, 36.03) = 4.29, *p* = 0.01, *η*^2^_partial_ = 0.26). Post hoc analysis revealed a time effect for the FDI muscle, during FDI pain (*F*(2.45, 29.35) = 4.11, *p* = 0.02, *η*^2^_partial_ = 0.26), but not during ECR pain (*F*(6, 72) = 0.81, *p* = 0.57, *η*^2^_partial_ = 0.06). Pairwise comparisons for the FDI MEPs showed that corticomotor excitability of the FDI was inhibited after 2 min (*p* = 0.01), 4 min (*p* = 0.029), and trending inhibition at 6 min (*p* = 0.074) compared to baseline (Figure 3). Individually, clear inhibition was found in 11 participants, whereas 2 participants exerted less inhibition or no change.

**Figure 4:**
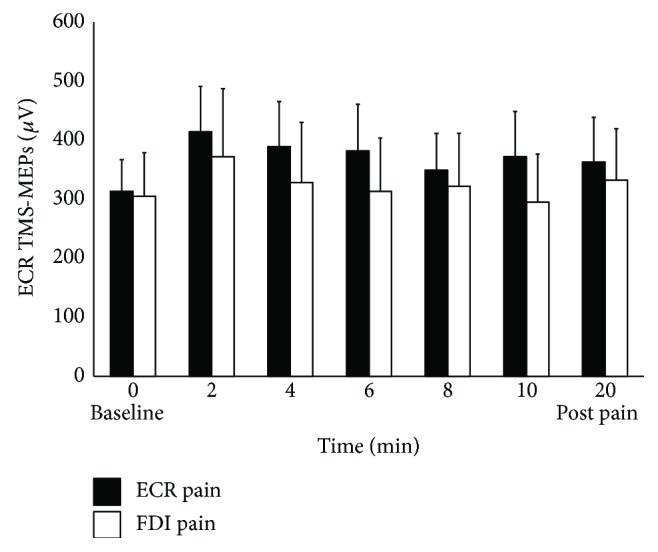
Mean (±SEM) MEPs of the ECR muscle following hypertonic saline injection into the ECR muscle (solid bars) and FDI muscle (open bars). Neither injection of hypertonic saline into the ECR muscle nor FDI muscle significantly altered the corticomotor excitability of the ECR muscle, at any time point compared to baseline. FDI: first dorsal interosseus; TMS: transcranial magnetic stimulation; MEPs: motor-evoked potentials.

For the ECR MEPs, the two-way RM ANOVA did not reveal a significant interaction (*F*(6, 72) = 0.74, *p* = 0.62, *η*^2^_partial_ = 0.06; Figure 4), but a significant main effect of pain site (*F*(1, 12) = 6.13, *p* = 0.03, *η*^2^_partial_ = 0.34).

### 3.4. Correlation Analyses

**Figure 5:**
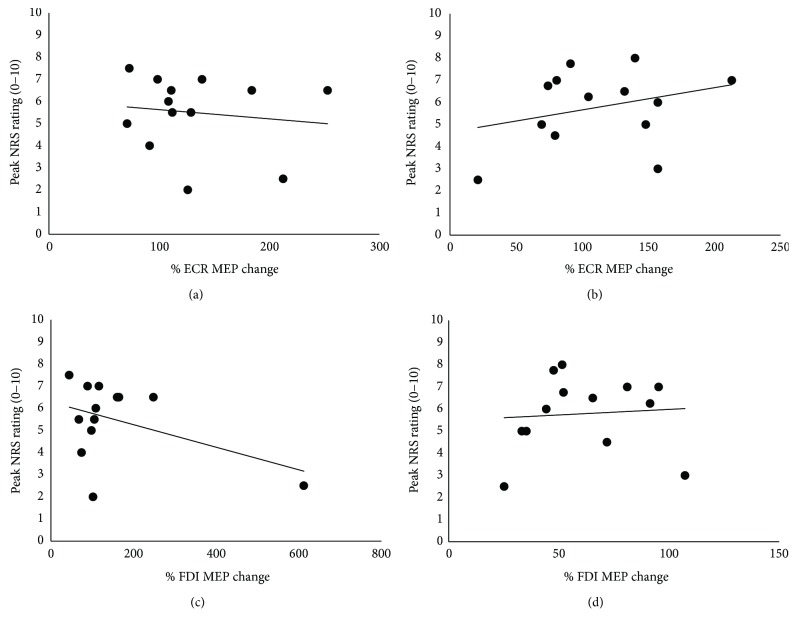
Correlations between percentage change in MEP magnitude versus peak pain intensity. No significant associations were found between percentage MEP changes from baseline at peak pain, with peak pain intensity for either the (a) ECR MEPs during ECR pain, (b) ECR MEPs during FDI pain, (c) FDI MEPs during ECR pain, or (d) FDI MEPs during FDI pain.

No significant associations were found between percentage MEP changes and peak pain intensity, for ECR MEPs during ECR pain (rho = −0.07, *p* = 0.83) (Figure 5(a)) or FDI pain (rho = 0.21, *p* = 0.49) (Figure 5(b)). Similarly, the FDI MEP percentage changes from baseline were not significantly associated with peak pain intensity during ECR pain (rho = −0.06, *p* = 0.84) (Figure 5(c)) or FDI pain (rho = 0.20, *p* = 0.51) (Figure 5(d)).

## 4. Discussion

The present findings show that the corticomotor excitability of the FDI muscle was reduced in response to hypertonic saline-induced FDI pain but not with ECR pain. In addition, corticomotor excitability of the ECR muscle did not change in response to hypertonic saline-induced ECR or FDI pain. These findings are contrary to the present hypothesis on simultaneous inhibition of both muscles. Further, an unexpected finding was that corticomotor excitability of the FDI muscle returned to baseline within 10 min after hypertonic saline injection, whereas previous studies have shown prolonged reduction of MEPs.

### 4.1. Reduction of Corticomotor Excitability in Response to Acute Muscle Pain

In this study, the FDI corticomotor excitability reduction in response to hypertonic saline-induced muscle pain is in line with previous studies which demonstrated a moderate-to-strong reduction in net corticomotor excitability during muscle pain [10, 11, 26]. Reduction of the FDI corticomotor excitability in association with pain has also been demonstrated following CO_2_ laser stimuli to the dorsum of the right hand [27] and capsaicin cream to the skin overlying the FDI [28]. Further, noxious heat applied to the lateral edge of the hand reduced the MEPs of FDI and opponens pollicis muscles [29]. Altogether, these studies support that noxious stimulation of the skin, regardless of modality, and muscle pain reduces corticomotor excitability of the FDI. The mechanisms underlying reduction of corticomotor excitability are still elusive. Peripheral M-wave amplitudes remain stable during pain in the tibialis anterior muscle, suggesting a central effect of pain on muscle activity [30]. Studies investigating spinal excitability, as measured by monosynaptic reflex activity (e.g., the Hoffmann reflex or cervicomedullary-evoked potentials), have reported inconclusive results and seem largely dependent on the muscle (distal versus proximal) [10, 26, 31]. These findings support the notion that acute experimental muscle pain predominantly causes inhibition at the cortical level during pain, without excluding spinal influences. Further, earlier evidence has demonstrated changes in gamma-aminobutyric acid and glutamate mediated inhibitory and facilitatory intracortical circuits in response to acute muscle pain, as assessed by paired-pulse TMS paradigms of hand musculature [14, 32]. However, studies have also reported that other muscles may react differently to pain. For instance, no change in corticomotor excitability of the masseter muscle was found following injection of hypertonic saline or application of capsaicin cream to the cheek [33]. Painful mechanical pressure increased corticomotor excitability of the brachioradialis muscle [34]. These findings, together with the lack of significant inhibition of the ECR corticomotor excitability in the current study, suggest that pain does not solely exert inhibitory effects on MEPs. The question then becomes why noxious stimulation of the FDI or hand, but not forearm musculature, exerts inhibitory effects on corticomotor excitability? It could be argued that the larger amplitudes of the FDI MEPs recorded in the current study offers a larger opportunity for reduction, whereas the lower amplitudes of the ECR MEPs may mask inhibitory effects on corticomotor excitability. Indeed, predetermined 500 *μ*V ECR MEPs at baseline have been shown to capture strong inhibition at pain resolve after hypertonic saline-induced pain [25]. Whether stimulus intensity is a key aspect of capturing ECR inhibition remains unknown, since this has not been investigated systematically. In respect to the current findings, baseline values for both the FDI and ECR muscles are in line with previously published literature, when stimulating at 120% RMT [14, 35], and as such is comparable in terms of corticomotor excitability changes. However, it cannot excludes that a similar pattern of ECR inhibition would occur if the intensity had been set to produce a predetermined MEP output at baseline, and needs further exploration.

### 4.2. No Simultaneous Pain Inhibition of Corticomotor Excitability of Hand and Forearm Muscles

This study demonstrates that the corticomotor excitability of two muscles adapts differently to the same experimental muscle pain model. The differential adaptation of corticomotor excitability of the FDI muscle versus the ECR muscle was contrary to expectations, given prior evidence which suggest that homotopic hand muscles (FDI and abductor digiti minimi) exhibit the same inhibition pattern during pain, independent of site of injection [10]. Furthermore, hypertonic saline injection into the flexor carpi radialis (FCR) produced a similar inhibition of FCR MEPs as seen for the hand muscles [10]. Most recently, this characteristic reduction was shown for the ECR corticomotor excitability over time in response to hypertonic saline [25]. Conversely, skin pain evoked by capsaicin cream application to the dorsum of the hand resulted in no significant reductions of the FCR or the flexor digitorum superficialis corticomotor excitability [36]. Together with the current findings, this challenges our understanding of how pain may influence corticomotor excitability. It is possible that MI inhibition is only one of many patterns of change that can occur in response to acute muscle pain. Whether these patterns of corticomotor excitability are a direct consequence of the pain or are also influenced by the function of the muscles involved [37] needs to be clarified. The present results show differential responses to muscle pain, supporting that different response patterns exist. Further research is warranted to fully appreciate the extent of possible different patterns and/or how muscle function influences corticomotor excitability changes in response to acute experimental muscle pain.

### 4.3. Time Course of MI Corticomotor Excitability in Response to Acute Muscle Pain

One common finding for most studies investigating pain and its effects on corticomotor excitability is that not only does pain affect the MEPs short term, but also outlasts the perception of pain [10, 11, 26]. This may indicate that corticomotor excitability changes are only partially driven by noxious input, such as that caused by hypertonic saline. In contrast and controversial to the earlier findings of a lasting inhibition of the corticomotor excitability [6], the results of the current study suggest that the reduction of FDI MEPs is susceptible to modulation during the acute pain period. It could be argued that other factors such as saliency of pain decreases over time and therefore allows a return-to-baseline for the motor output, however, since the participants still perceived pain after 10 min, this is unlikely to be the only explaining factor. Further, since the participants were asked to verbally rate their pain intensity during the pain and TMS application, both motor activation due to speech [38] together with attentional factors [39] may have had an impact on corticomotor excitability. Nonetheless, it is important to note that participants always rated in-between two TMS stimulations (both muscles), but we cannot rule out possible carryover effects. Future studies may explore the impact of speech (pain rating) or attention towards pain intensity while temporally profiling corticomotor excitability during pain, to understand its potential contribution to the current findings.

### 4.4. Differential Modulation of Corticomotor Excitability and Its Relation to Functional Outcomes

Most studies have investigated isolated muscles (most commonly effector muscles) in pain, when assessing functional changes, such as motor control performance or learning. For instance, during training of a novel tongue-protrusion task, it was shown that capsaicin cream applied to the tongue reduced the gains in excitability of the tongue MI that would otherwise occur during training in a pain-free state, however, gains in performance were still evident [9]. A later study corroborated that pain can interfere with corticomotor excitability increases, but not necessarily acquisition of the motor skill [40]. It has further been reported that if pain affects acquisition of movement patterns during training, these altered movement patterns may persist 24 hours later [41]. Additionally, neck training in the presence of acute hypertonic saline-induced pain may induce long-lasting inhibition of corticomotor excitability [42]. Conversely, other studies have reported that different topical pain paradigms such as capsaicin [40, 43–45] or heat [46] do not alter acquisition during performance of different motor tasks, but retention may be enhanced [45]. As such, a large body of contrasting evidence is available, and the interaction between pain and motor acquisition/retention remains controversial. Further research is needed to understand the impact of different effector muscles in relation to pain and its effects on motor learning outcomes.

In support of the current findings, differences in corticomotor excitability responses between the FDI and ECR have been described previously but in relation to voluntary contractions [47] and median nerve stimulation [48]. For instance, voluntary contractions spanning 0–100% MVC revealed a large difference in the level of contraction needed for saturation of the MEPs due to TMS [47]. This finding was ascribed to differences in corticomotoneuronal connections between distal hand musculature and proximal forearm musculature [47]. This also suggests that hand musculature is more readily susceptible to neuroplastic adaptations to external stimuli than that of more proximal muscles such as the ECR muscle. These lines of evidence suggest that (1) corticomotor excitability changes may play a pivotal role during motor learning and (2) the corticomotor inhibition may serve to prevent acquisition of harmful movement patterns [7] but the overall response could be dependent on the function of each muscle [49]. Since few studies have investigated the possibility of preventing or reversing corticomotor inhibition [25] and the functional outcome of this, the potential of improving motor relearning paradigms remains unknown.

### 4.5. Limitations

In this study, only one optimal scalp position or the hotspot was tested in each session as we aimed to understand the change in cortical excitability throughout pain duration. The pain duration in this study was relatively short thus limiting the feasibility to assess cortical excitability of the ECR and FDI at each respective hotspot. The benefit of using an acute experimental pain model such as hypertonic saline injections is that it allows for standardization of time and location of the pain [8, 50–52]. The disadvantage is the short-lasting pain evoked by hypertonic saline which limits our ability to infer about clinical relevance. In general, small sample sizes compromise generalizability and should be considered. Since no control group (i.e., isotonic saline injection) was enrolled, it cannot be excluded that the reduction in FDI corticomotor excitability is due to a placebo effect. Nonetheless, earlier evidence suggests that a nociceptive input (such as the one experienced from hypertonic saline injection) is pivotal for the reduction in corticomotor excitability [10, 12, 26]. Further, MEP amplitudes are reliable as a repeated measure [26, 53], thereby excluding time as an explanatory factor for MEP reduction. Of note, we opted not to include measures of spinal motoneuronal excitability, since this was outside the scope of the current study. Therefore, we are not able to rule out a spinal influence on the corticospinal excitability reduction (denoted as corticomotor excitability) in response to hypertonic saline. Nonetheless, studies investigating H-/F-wave amplitudes in response to pain have shown that spinal motoneuronal excitability does not change [28] or is reduced after peak pain [10], during acute experimental pain.

## 5. Conclusions

The current study demonstrates that two topographically overlapping muscle representations within the MI adapt differently to hypertonic saline-induced muscle pain. This suggests a nonuniform effect of pain on corticomotor excitability across a distal and a proximal muscle. The lack of prolonged inhibition of FDI corticomotor excitability warrants further investigations on how different methodologies may provide insight into the neuroplasticity of corticomotor excitability.
